# A multi-class driver behavior dataset for real-time detection and road safety enhancement

**DOI:** 10.1016/j.dib.2025.111529

**Published:** 2025-04-04

**Authors:** Arafat Sahin Afridi, Arafath Kafy, Ms. Nazmun Nessa Moon, Md. Shahriar Shakil

**Affiliations:** Department of Computer Science and Engineering, Daffodil International University, Dhaka, Bangladesh

**Keywords:** Driver image dataset, Image annotation, Data augmentation, Vehicle classification, Convolutional neural network, Computer vision

## Abstract

This paper introduces a novel dataset designed to support the development of AI-driven driver monitoring systems. The dataset captures real-world driver behaviors under diverse driving conditions, including private vehicles and public buses, in Dhaka, Bangladesh. It comprises 7286 high-resolution images categorized into five behavioral classes: Safe Driving, Talking on the Phone, Texting, Turning, and Other Distracting Behaviors. The dataset reflects natural variations in driver behavior, such as different lighting conditions, angles, and vehicle types, making it highly applicable to real-world scenarios. By providing a comprehensive and annotated dataset, we aim to support the development of intelligent transportation systems and contribute to reducing accidents caused by distracted driving. The dataset is publicly available and can be used to train and evaluate machine learning models for real-time driver behavior detection.

Specifications Table

**Table:** 

Subject	Computer Science
Specific subject area	Driver Behavior Analysis, Computer Vision, Real-Time Detection, Road Safety Monitoring
Type of data	JPEG
Data collection	This data was collected in October 2024 for research purposes. The photos were taken in Savar, Dhaka, Bangladesh, using two separate personal cell phone cameras in both private vehicles and public buses.
Datasource location	**Location:** Daffodil International University, Birulia. **Zone:** Savar, Dhaka-1216 **Country:** Bangladesh **Latitude:** 23.5234° N, **Longitude:** 90.1915° E
Data accessibility	**Repository name:** Zenodo Data **Data identification number:**10.5281/zenodo.14908802 **Direct URL to data:**https://zenodo.org/records/14908802

The main significance of this dataset is that it can help in the study and development of intelligent systems to improve driver safety by immediately detecting dangerous behavior. Below are some of the key applications for the dataset:

• **Improving road safety**: Using this data can enable systems that monitor driver behavior and alert drivers to potentially dangerous behaviors, such as texting, making phone calls, or falling asleep while driving. Installing these technologies in cars can reduce the number of collisions caused by distracted drivers.
• **Real-time monitoring**: The dataset can be used to create real-time driver monitoring systems that can reduce roadside distractions, enforce safe driving habits, and assist in traffic control. This has implications for improving safety, optimizing traffic flow, and reducing collisions caused by human error.
• **Machine learning and computer vision**: This dataset can be used to train and evaluate deep learning models, particularly in the areas of image recognition and classification. Researchers can use CNNs such as VGG16, ResNet50, InceptionV3, and Xception to compare and evaluate different approaches to identify specific driver behaviors. This allows them to learn more about the applicability and effectiveness of each model in real-world scenarios.
• **Public Safety**: The dataset supports both specific applications and broader efforts to improve public safety. Roads become safer for all users when law enforcement and transportation agencies detect unsafe driving in real time to monitor and enforce driving regulations.

## 1 Background

Road traffic accidents account for over 1.35 million fatalities annually, according to the World Health Organization (WHO) [1]. Among the primary causes is distracted driving, characterized by activities such as texting or phone usage, which diverts attention from the road [2]. Developing countries like Bangladesh face heightened challenges due to increasing vehicle density [3], inadequate enforcement, and diverse road conditions [4,5]. This study addresses these challenges by introducing a novel dataset tailored for real-world driver behavior detection. Unlike prior research that often relies on simulated or controlled data, our dataset captures diverse real-world conditions, ensuring applicability in practical scenarios [6]. Several driver behavior datasets exist, such as the StateFarm Driver Distraction dataset [7], which primarily consists of staged environments. Another dataset, Wawage et al.'s Smartphone Sensor Dataset for Driver Behavior Analysis [5], captures sensor-based data but lacks real-world image-based representations. Our dataset differs by focusing on real-world [[8], [9]], naturalistic driving scenarios, ensuring its applicability in real-time AI-driven monitoring systems. Furthermore, studies such as Lin et al. [[10], [11]] and Zhang et al. [12] highlight the importance of real-world driving datasets for improving AI-based detection systems.

Driver distraction is one of the leading causes of road accidents, posing a significant threat to public safety [13]. While AI-based driver monitoring systems have the potential to mitigate this issue, their effectiveness depends on high-quality training data [[14], [15]]. Existing datasets often rely on simulated environments or controlled conditions, which fail to capture the complexities of real-world driving scenarios. This gap limits the accuracy and generalizability of AI models in practical applications. To address this challenge, we introduce a new dataset specifically designed to reflect real-world driving conditions. Captured in private vehicles and public buses under diverse lighting and environmental factors, this dataset provides a valuable resource for developing AI-driven monitoring systems to detect and prevent distracted driving in real time.

## 2 Data Description

The dataset comprises 7286 high-resolution images captured in Ashulia, Dhaka, Bangladesh, under real-world driving conditions, encompassing both private vehicles and public buses. Data collection was conducted across a variety of settings, including urban roads and highways, to ensure diversity in traffic density, vehicle types, and driver behaviors. To enhance the dataset's variability and realism, images were captured from multiple angles and under different lighting conditions, such as daylight, low light, and nighttime.

The study involved 178 drivers aged 20–50 years, representing a diverse demographic to ensure the dataset's generalizability across different age groups and driving experiences. Data collection spanned over a period of 30 days, with each driver being monitored for approximately 1–2 hours per day. Images were captured under varying lighting conditions and angles to simulate a wide range of real-world driving scenarios. Each image was manually annotated into one of five behavior classes, ensuring accurate labeling for training and evaluation purposes. This approach ensures that the dataset reflects the complexities and nuances of real-world driving, making it a valuable resource for developing AI-driven driver monitoring systems (Table 1).

**Table 1:** Description of driver behavior detection dataset.

Class Name	Description	Visualization
Safe Driving	Driving with complete focus, no distractions. The driver maintains good posture and a firm grip on the steering wheel while paying close attention to the road.	
Talking phone	The driver is using the phone in their hand or holding it to their ear while making a phone call. This conduct is a frequent contributor to accidents since it takes the driver's focus away from the road.	
Texting phone	The driver is texting while driving, which significantly diverts their attention from the road.	
Turning	The driver is turning the car, requiring full attention to ensure the maneuver is performed safely.	
Others	This category covers various distracting behaviors, such as talking to passengers, eating, drinking, or sleeping while driving. These behaviors are common in real-world driving scenarios and significantly increase the risk of accidents by reducing the driver's awareness and reaction time.	

### 2.1 Dataset statistics

• Safe Driving: 1679 images
• Turning: 1343 images
• Talking on the Phone: 1513 images
• Texting on the Phone: 1561 images
• Others: 1190 images

Each image has a resolution of 400 × 600 pixels, ensuring consistency for machine learning applications. The dataset includes variations in lighting, vehicle type, and driving conditions, reflecting real-world scenarios.

## 3 Experimental Design, Materials and Methods

### 3.1 Camera specification

The dataset was collected using two smartphones:

• **iPhone 11**: Equipped with a dual-lens camera system (12MP wide-angle and 12MP ultra-wide-angle lenses) and features such as optical image stabilization, True Tone Flash, and Smart HDR for high-quality images in various lighting conditions.
• **Moto Edge 50 Fusion**: Features a 50MP primary lens and a 13MP ultra-wide lens, with additional features such as panorama mode, HDR support, and LED flash for flexible photography in different environments.

These devices were chosen for their ability to capture detailed and high-quality images under diverse driving conditions.

The expansion process significantly expanded the data set by providing each class with a set of unique image variations. These enhanced photos maintained their semantic meaning while capturing the natural variations in driver behavior as well as external elements such as lighting and angle changes. This expanded dataset was used to train deep learning models, improving their performance in real-world scenarios and generalization.

### 3.2 Preprocessing steps

To ensure the dataset's suitability for machine learning tasks, the following preprocessing steps were applied:

• Noise Reduction: Impulse noise was removed using non-linear median filtering to enhance image clarity.
• Contrast Enhancement: Histogram equalization was used to adjust for irregular lighting and improve contrast.
• Image Resizing: All images were resized to a fixed dimension of 224 × 224 pixels to meet the input requirements of deep learning models.

### 3.3 Data augmentation techniques

Online data augmentation techniques were applied during the training process to increase the dataset's variability and simulate real-world scenarios. These techniques included:

• Random Rotation (up to 15°)
• Horizontal Flipping
• Brightness Adjustment
• Random Cropping

Augmentation was performed online to avoid introducing bias and to ensure that the dataset remains suitable for unbiased evaluation.

**Fig. 1:**
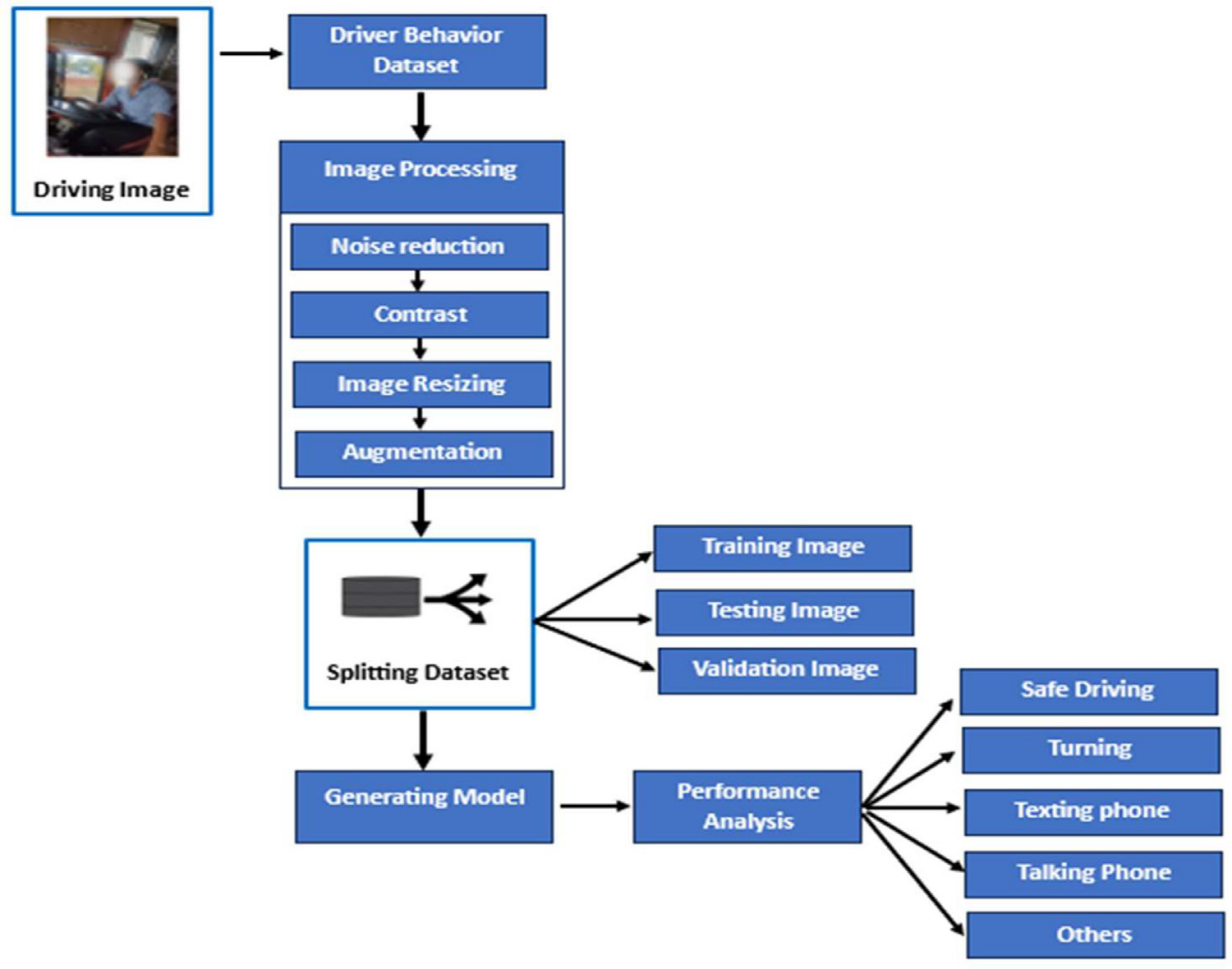
Working procedure.

**Data Augmentation:** The dataset was divided into five classes: Safe Driving, Turning, Texting, Talking on Phone, and Other Distractions. Each class was saved in a separate folder. Data augmentation was used to increase the variability of the dataset by simulating various real-world scenarios using techniques such as rotating, flipping, zooming, brightness adjustment, and cropping. The expansion process is designed to automatically preserve the original captions on the images. Extended images were stored in the same folder as their corresponding original class to ensure that they inherited the correct labels without requiring additional manual intervention (Fig. 1).

**Fig. 2:**
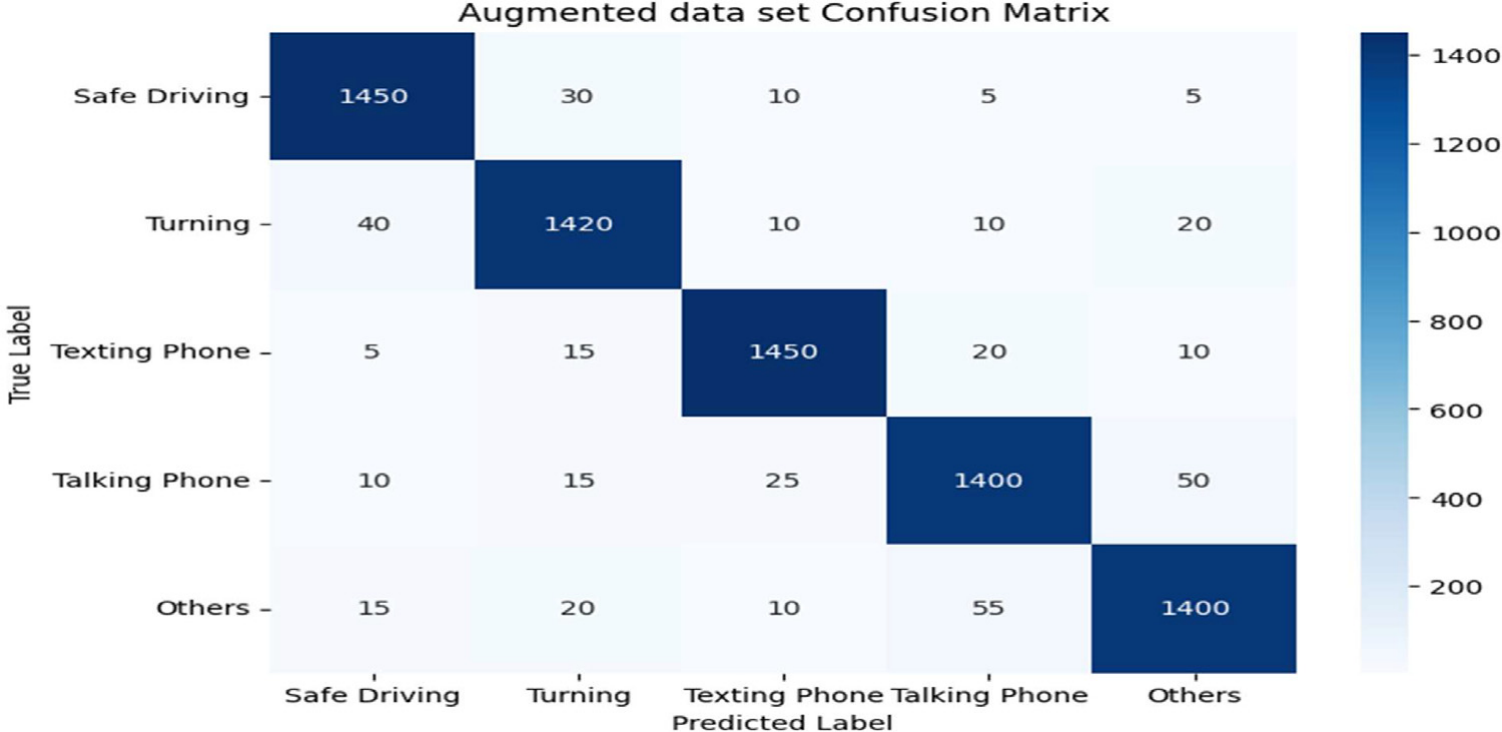
Confusion matrix.

Fig. 2 is a confusion matrix for an augmented dataset, showing the performance of a classification model across five categories: Safe Driving, Turning, Texting Phone, Talking Phone, and Others. The numbers in each cell represent the count of actual vs. predicted labels for each category. For example, the top-left cell shows that 1450 instances of ``Safe Driving'' were correctly predicted as ``Safe Driving,'' while 30 instances were incorrectly predicted as ``Turning.'' This matrix allows for analysis of where the model performs well and where it struggles in classifying different driving behaviors.

### 3.4 Data augmentation

The dataset was divided into five classes: Safe Driving, Turning, Texting, Talking Phone and Other. Each class was saved in a separate folder. Data augmentation was used to increase the variability of the dataset by simulating various real-world scenarios using techniques such as rotating, flipping, zooming, brightness adjustment, and cropping. Each of the data set's five classes - safe driving, turning, texting phone, talking phone and others - was stored in a separate folder. Data augmentation was used to increase the variability of the dataset by simulating various real-world scenarios using techniques such as rotating, flipping, zooming, brightness adjustment, and cropping. The expansion process is designed to automatically preserve the original captions on the images. Extended images were stored in the same folder as their corresponding original class to ensure that they inherited the correct labels without requiring additional manual intervention.

The expansion process significantly expanded the data set by providing each class with a set of unique image variations. These enhanced photos maintained their semantic meaning while capturing the natural variations in driver behavior as well as external elements such as lighting and angle changes. This expanded dataset was used to train deep learning models, improving their performance in real-world scenarios and generalization (Table 2).

**Table 2:** Augmented images of Driver behavior detection dataset.

## 4 Contribution of this dataset

This dataset is unique as it captures real-world driving conditions across diverse environments, including private vehicles and public buses, ensuring a comprehensive representation of driver behaviors. Unlike simulated datasets, it reflects natural variations such as different lighting conditions, angles, and vehicle types, making it highly applicable for real-world scenarios. The dataset has significant potential applications, particularly in the development of AI-driven driver monitoring systems for real-time behavior detection. By enabling more accurate recognition of distracted driving, it can contribute to traffic safety improvements and accident prevention. Compared to existing datasets like the StateFarm Driver Distraction dataset, which primarily consists of staged environments, this dataset offers a more authentic representation of driver behaviors, enhancing its usability for practical deployment in intelligent transportation systems.

## 5 Impact of this dataset

This dataset presents several unique impacts:

1. **Real-World Applicability**: Captures natural driving behaviors in dynamic urban conditions.
2. **Diverse Data Collection**: Includes both private and public transport scenarios.
3. **High-Quality Annotations**: Expert-validated labels ensure robust dataset reliability.
4. **Facilitates AI Development**: Enables researchers to develop and benchmark real-time driver monitoring models.

The dataset supports applications in autonomous vehicle safety, traffic surveillance, and intelligent driver assistance systems, significantly contributing to road safety research.

## 6 Limitations

While the dataset captures diverse real-world driving conditions, its geographic focus on Dhaka, Bangladesh, limits its global applicability. To improve generalization, future expansions should include data from different regions, road infrastructures, and demographic groups, ensuring a broader representation of driving behaviors. Additionally, the current dataset consists of five behavior classes, but future iterations should incorporate more complex driving behaviors, such as lane-changing, aggressive driving, and drowsiness detection, to enhance its applicability in real-world driver monitoring systems detection.

• **Geographic Scope**: The dataset was collected exclusively in Dhaka, Bangladesh, which may limit its generalizability to different geographic regions. Driving behaviors, environmental conditions, and road structures vary significantly across countries. Expanding data collection to include different urban and rural settings worldwide will improve the dataset's adaptability and robustness.
• **Behavioral Complexity**: While the dataset covers common distracted driving behaviors, it does not yet include more nuanced actions such as **drowsiness, aggressive driving, and reckless lane changes**. Future expansions should incorporate these additional classes to improve the dataset's effectiveness for advanced driver monitoring applications.
• **Limited Sensor Modalities**: The dataset primarily relies on smartphone cameras for image collection. The absence of depth sensors, infrared cameras, or multi-modal data sources (such as LiDAR or EEG signals) may limit its applicability in low-light conditions or drowsiness detection. Future research should explore integrating these advanced sensing technologies for a more comprehensive driver monitoring system.

## 7 Future directions

To further enhance the effectiveness and applicability of AI-driven driver monitoring systems, several key improvements can be made. First, the dataset can be expanded to include additional driver behaviors, such as aggressive driving, drowsiness, and lane-changing, as well as more diverse environmental conditions, including adverse weather and varying road types. Second, the development of lightweight deep learning models optimized for real-time deployment on mobile and edge devices is essential to ensure efficient in-vehicle monitoring with minimal computational resources. Lastly, integrating multimodal data, such as audio cues (e.g., driver alerts, in-car conversations) and video streams, can significantly enhance detection accuracy by providing a more comprehensive understanding of driver behavior. These advancements will contribute to the development of more robust and practical intelligent transportation systems aimed at improving road safety.

## 8 Conclusion

This dataset provides a real-world representation of driver behaviors and can be instrumental in developing AI-driven driver monitoring systems. Unlike staged datasets, it captures actual driving conditions, making it a valuable resource for traffic safety research. Future work should aim to expand data collection across multiple geographic regions and incorporate additional behavior classes such as drowsiness and aggressive driving.

## Ethics Statement

In accordance with ethical standards, all participants provided informed consent before data collection. To ensure privacy and confidentiality, no identifiable or personal information was collected during image capture. With the help of the drivers, the photos were taken in a controlled and safe manner, with no one injured.

## CRediT Author Statement

**Arafat Sahin Afridi:** Conceptualization, Data curation, Methodology, Visualization, Validation. **Arafath Kafy:** Writing – original draft, Writing – review & editing, Methodology, Data curation. **Ms. Nazmun Nessa Moon:** Supervision, Writing – review & editing. **Md. Shahriar Shakil:** Co-Supervision Methodology, Data curation.

## Data Availability

doi: 10.5281/zenodo.14908802.Multi-Class Driver Behavior Image Dataset (Original data).
